# Preliminary Analysis of the Glycolipid Profile in Secondary Brain Tumors

**DOI:** 10.1155/2022/4293172

**Published:** 2022-11-22

**Authors:** Alina-Florina Serb, Cristina Novaconi, Marius Georgescu, Maria Puiu, Alis Dema, Robert Onulov, Eugen Sisu

**Affiliations:** ^1^Victor Babes University of Medicine and Pharmacy Timisoara, Biochemistry and Pharmacology Department, Biochemistry Discipline, Eftimie Murgu Sq. no. 2, 300041, Timisoara, Romania; ^2^Victor Babes University of Medicine and Pharmacy Timisoara, Faculty of Medicine, Eftimie Murgu Sq. no. 2, 300041, Timisoara, Romania; ^3^Victor Babes University of Medicine and Pharmacy Timisoara, Functional Sciences Department, Physiology Discipline, Eftimie Murgu Sq. no. 2, 300041, Timisoara, Romania; ^4^Center of Immuno-Physiology and Biotechnologies (CIFBIOTEH), “Victor Babeș” University of Medicine and Pharmacy Timișoara, Eftimie Murgu Sq. no. 2, 300041 Timișoara, Romania; ^5^Victor Babes University of Medicine and Pharmacy Timisoara, Functional Sciences Department, Genetics Discipline, Eftimie Murgu Sq. no. 2, 300041, Timisoara, Romania; ^6^Victor Babes University of Medicine and Pharmacy Timisoara, Functional Sciences Department, Morphopathology Discipline, Eftimie Murgu Sq. no. 2, 300041, Timisoara, Romania

## Abstract

Glycosphingolipids (GSLs) play numerous roles in cellular processes, including cell proliferation, apoptosis, inflammation, and cell signaling. Alteration of the GSLs metabolism leads to the accumulation of particular species of GSLs, which can lead to various pathologies, including carcinogenesis and metastasis; in essence, all neoplasms are characterized by the synthesis and aberrant organization of GSLs expressed on the cell surface. Secondary brain tumors make up the majority of intracranial cancers and generally present an unfavorable prognosis. In the present work, a native GSL mixture extracted and purified from a secondary brain tumor with primary pulmonary origin was obtained through extraction and purification and analyzed by MALDI TOF mass spectrometry. Research in the field of lipidomics could offer new data for the understanding of brain tropism and metastatic pathways, by studying the glycolipid molecules involved in the process of metastasis in general and in the production of brain metastases in particular. This could shed new light on the pattern of lipid glycosylation in secondary brain tumors, with a great impact on the effectiveness of cancer therapies, which could be adapted to the specific molecular pattern of the tumor.

## 1. Introduction

Metastatic, or secondary, cerebral cancer occurs when a neoplasm spreads to the brain from another part of the body. It is approximately ten times more common than primary cerebral neoplasms—those which develop in the brain from the start. The risk of developing a metastatic cerebral malignancy is dependent on the type and the histological grade of the primary tumor.

Metastatic brain tumors most often originate from malignancies in the lungs, breasts, colon, kidneys, or skin. Of these, lung neoplasm is one of the more aggressive types of cancer, increasingly common worldwide, and known for its susceptibility to metastasize to multiple organs, resulting in a poor prognosis. Compared with other primary cancers, in which cerebral metastases are usually a late complication, in lung neoplasms, intracranial metastases are developed relatively early and are often accompanied by neurological symptoms on initial diagnosis [1, 2]. All types of malignant cells demonstrate changes in their glycosylation at the cell surface, and many glycosyl epitopes represent tumor-associated antigens. When highly expressed, some of those epitopes promote invasion and metastasis and can lead to a worse prognosis and shorter survival rates [2].

Glycosphingolipids (GSLs), which are the glycosides of ceramide (Cer) are a structurally and functionally diverse sphingolipid subclass ubiquitously distributed among all eukaryotic species with more prevalence in the nervous system, and also found in some bacteria. As with other carbohydrates and glycoconjugates, the potential for variation of GSL glycan structure is enormous, especially when one includes noncarbohydrate modifications such as sulfation, acetylation, fatty acid acylation, cyclic fatty acid acylation, and methyl or other etherifications; interpolation of phosphate or myo-inositol-1-phosphate. To date, more than 180 gangliosides (acidic GSLs) species with different glycan headgroups have been identified in vertebrate tissues [3, 4].

Glycosphingolipids (GSLs) are ubiquitous components of cell membranes that can act as mediators of cell adhesion and signal transduction and can be used as cell type-specific markers. They are associated with different mammalian diseases including lung, breast, prostate, and ovarian cancer, as well as cerebral neoplasms, and therefore the reliable identification of ganglioside isomers is essential for biomarker discovery studies. Tumors display changes in ganglioside expression on cell membranes. These changes lead to modulation of cell signaling pathways and the acquisition of chemoresistant properties [5]. Gangliosides could be shed from tumor cells into plasma, the serum or could be secreted via exosomes, bind to the surface of normal cells, and change the lipid composition of raft domains. They often integrate cells surrounding tumors' microenvironment such as fibroblasts, endothelial, or immune cells. Gangliosides are involved in tumor-associated T-cell activation [5, 6]. Altered glycosphingolipid (GSL) glycosylation of the cell surface membrane during malignancy can affect cell recognition, adhesion, and signal transduction and is found to reflect: (1) incomplete synthesis with or without precursor accumulation, (2) neosynthesis, (3) increased sialylation, and (4) increased fucosylation [7]. The upregulation of fucosyltransferases in neoplasm was shown to cause a higher degree of fucosylation in malignant tissues, and it was proposed that the increase in the fucosylation for GSLs was an early event in cancer [8, 9].

During the last three decades, numerous mass spectrometry (MS)-based methods have been developed that were specifically tailored to obtaining insight into the different aspects of glycoconjugate structures, including GSLs. Among these, matrix-assisted laser desorption/ionization (MALDI) MS have been found to be especially powerful for determining: (a) molecular masses of oligosaccharides up to the range of more than 30 sugar units, (b) sequence and pattern branching, and (c) structure of the aglycon [10].

Mass spectrometry is now an indispensable tool for lipid analysis and is arguably the main driving force in the lipid research renaissance. In its various forms, mass spectrometry is uniquely capable of resolving the extensive compositional and structural diversity of lipids in biological systems. Furthermore, it provides the ability to accurately quantify molecular-level changes in lipid populations associated with changes in metabolism and environment, bringing lipid science to the “omics” age [11].

The use of MALDI mass spectrometry entails mixing the analyte with an acidic matrix and drying a low microliter volume on a metal target, forming crystals. Subsequent irradiation with pulsed laser light creates a molecular plume containing ionized analyte and matrix molecules that may be mass analyzed. MALDI sample preparation methods are relatively tolerant of salts and other nonsurfactant additives or contaminants, with the result that data is obtained with minimal sample workup [12].

Our study presents the profile obtained by MALDI-TOF MS of a complex GSL extract acquired following a laborious and careful process of isolation and purification from a secondary brain tumor with pulmonary origin. Our findings could offer new data on the pattern of lipid glycosylation and provide insights into the mechanisms of cancer development and therapy targeting glycosylation.

## 2. Materials and Methods

### 2.1. Biological Material

The investigated brain tumor sample, 0.63 g, was obtained after surgical resection from a male patient, aged 66, and was defined as a secondary brain tumor (metastasis) originated from a NSCLC (non-small-cell lung cancer)-type, namely, poorly differentiated pulmonary adenocarcinoma. The mass of the purified raw mixture of GSLs, which was analyzed by MALDI TOF MS, weighed 4.31 mg. The study was approved by the Ethical Committee of the University of Medicine and Pharmacy “Victor Babes” Timisoara, Romania (Approval code no. 41) and the Ethical Committee of the Regional Hospital “Pius Branzeu” Timisoara, Romania (Approval code no. 04).

### 2.2. Reagents

All reagents were of analytical grade and were obtained as follows: sodium hydroxide, phenol (>99%) and sulfuric acid 95-97%, 2,5-DHB were purchased from Merck; chloroform, methanol, acetonitrile (all chromatographic grade), Sephadex G-25 (Fine) from Sigma-Aldrich (St. Louis, MO, USA).

### 2.3. Extraction and Purification of Lipids

In order to isolate the raw mixture of acidic GSLs from cerebral tumor tissue, the sample (0.63 g) was subjected to successive steps of extraction and purification [13, 14].

First, the sample was weighed and subjected to homogenization in a blender in the presence of ice, obtaining a homogenate of about 10% (dry substance content). Then, the homogenate undergoes extraction twice at a temperature of less than 5°C with a mixture of solvents (CHCl_3_ : MeOH : H_2_O = 1 : 2 : 0.75, v : v : v), followed by steps of partition and repartition by adding MeOH and H_2_O to a volume ratio of 1 : 1 : 1. The upper phases (MeOH-H_2_O) containing polar GSLs are separated from the lower ones and brought to dryness in a rotary evaporator (Speedvac SPD121P ThermoScientific). Further, the sample was purified by gel filtration [14]. Separation control is done by TLC. The tubes containing the fraction of interest are reunited, and the solvent is removed by entrainment with N_2._ Finally, the raw mixture is dried in a desiccator over P_2_O_5_.

### 2.4. Sample Preparation for Mass Spectrometry

The mass of the native mixture of GSL extract obtained after extraction, separation, and purification was 4.31 mg. In order to analyze by MS, stock solutions were made using 99.8% purity methanol as solvent. In order to prepare the stock solutions, the sample was dissolved in 2 ml of methanol. These were then vortexed and centrifuged at different speeds and for different time intervals—the optimal parameters observed that allowed for sedimentation of all impurities were 2000 rpm for 20 min. For the MS analysis, multiple dilutions with 99.8% methanol were made in order to obtain a continuous and constant infusion necessary for the ionization of the species of interest and for obtaining an optimal signal.  Table 1 shows the dilutions prepared and the concentrations obtained for the sample of the metastatic brain tissue. We determined that the spectrum obtained for the tumor brain tissue sample, corresponding to the 1: 700 dilution, was of the best quality, and therefore the screening and structural analysis experiments were be performed using this dilution, and the concentration of the methanolic solution of the native mixture of GSLs that will be further analyzed by MS will be of 2.45 pmol/*μ*L.

### 2.5. Mass Spectrometry Assay

MALDI-TOF mass spectra of the samples were recorded by using a Bruker Ultraflextreme MALDI-TOF workstation controlled by FlexControl (Bruker Daltonics, Bremen, Germany) equipped with a Smartbeam II laser (Bruker Daltonics) of 355 nm and operating in negative mode. The following settings were used: laser frequency, 2,000 Hz; smart beam, ‘4_large'; sample rate and digitizer settings, 1.25 GS/s; accelerator voltage, 20.07 kV; extraction voltage, 18.87 kV; lens voltage, 5.58 kV; and delayed extraction, 250 msec. 1,000 laser shots were used for each individual spectrum, and a minimum of 10 individual spectra was cumulated and saved. MS spectra were calibrated externally using a ganglioside mixture (ammonium salt, bovine brain) Calbiochem, Merck KGaA, Darmstadt, Germany, which results in optimal spectra for both MS and MS/MS analysis. FlexAnalysis, version 3.3 (Bruker Daltonics) was used for data processing. The desalted sample (1.0 mL) was spotted on MTP 384 polished steel BC targets (Bruker Daltonics). Samples were overlaid with 2,5-Dihydroxybenzoic acid (2,5-DHB) (1.0 mL) and left for drying (dried droplet method). Matrix solubilization procedure included dissolution of 2,5-DHB (20 mgxmL^−1^) in a mixture of water/methanol/acetonitrile (6 : 1 : 3, vol/vol/vol) with/without the addition of 0.1% HCOOH or trifluoroacetic acid (TFA), respectively.

## 3. Results

### 3.1. TLC Control of Lipid Mixtures from Secondary Brain Tumor Tissue

Separation by TLC was tested and optimized to separate GSL fractions. Thus, numerous experiments were performed to separate and visualize the standard mixtures of gangliosides with several solvent mixtures used for elution and with several types of stationary phases and systems used for visualization of the separated fractions. Several supports were tested as stationary phase: silica gel F254, cellulose, polyamide, while for elution several mixtures of solvents have been tried: methanol: chloroform: CaCl_2_ in a concentration of 2 and 3%, in a volumetric ratio of 40: 50 : 13, and methanol: chloroform: water in different volumetric ratios: 35 : 55 : 10, 30 : 60 : 5, 40 : 50 : 10, etc. The results obtained highlight the considerably different efficiency of these systems in separating the glycolipid fractions from the mixtures. In this respect, the methanol: chloroform: water system (30 : 60 : 5) did not allow a satisfactory migration of the separated fractions, and the methanol: chloroform: 3% CaCl_2_ system (40 : 50 : 13) did not make the complete separation of glycolipid fractions possible. The optimal result in migration and separation was obtained using the elution system methanol: chloroform: water (40 : 50 : 10). In stages of highly dilute ganglioside solutions, these cannot be directly detected by the use of the resorcinol acid solution (ethanolic solution of H_2_SO_4_ (20%)). A much more sensitive variant, which has been tested and used to visualize GSLs, is the use of the reagent with ammonium molybdate and cerium sulfate (+4), for which it is not necessary to keep the plate in the oven to complete the reaction.

**Figure 1:**
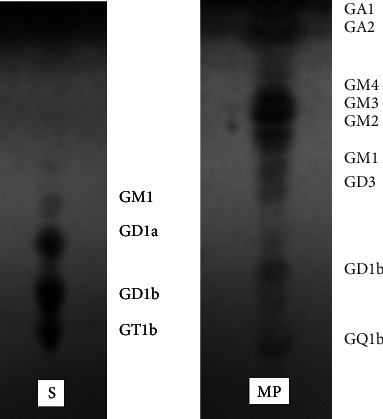
TLC chromatogram of acidic GSLs. Line 1: standard gangliosides mixture; Line 2: gangliosides mixture isolated from brain tumor sample; Elution: MeoH : CHCl_3_ : H_2_O = 40 : 50 : 10 (v/v/v); visualization: Ce(SO_4_)_2_: (NH_4_)_6_Mo_7_O_24_.4H_2_O : H_2_SO_4_ : H_2_O = 1 : 5 : 10 : 90 (w) then, 5 min to 120°C.

Figure 1 shows the TLC chromatogram obtained after the collection of GSL fractions separated by gel filtration chromatography during the previous step of the purification procedure.

### 3.2. MS Screening of Lipid Mixtures from Secondary Brain Tumor Tissue

Prior to MS analysis, initial work was conducted to determine the optimal matrix preparation for the analysis of lipid native mixtures from brain tumor tissue. A 2,5-DHB matrix was prepared in a mixture of water, methanol, and acetonitrile 6 : 1 : 3 (vol/vol/vol) and was used with or without the addition of formic acid and trifluoroacetic acid (TFA) to evaluate the quality of mass spectra with respect to the resolution, the signal to noise ratio and the low matrix interference peaks. DHB matrix used without the addition of TFA and formic acid produced the best quality mass spectra, and therefore, it was used further for sample ionization. Various concentrations of the 2,5-DHB matrix were tested in the range of 10-30 mg/ml, and the optimum results were obtained with a concentration of 20 mg/ml.

**Figure 2:**
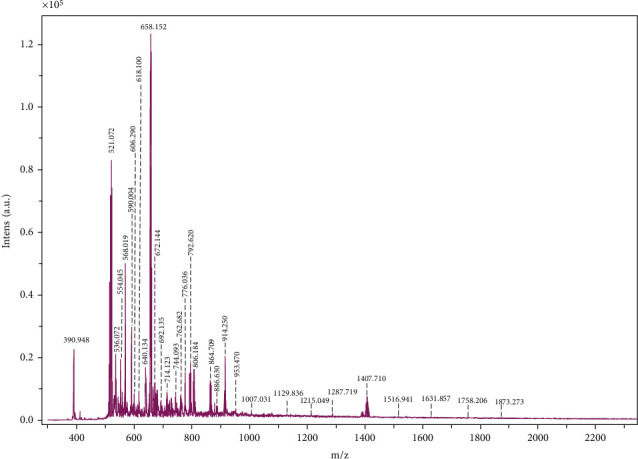
MALD QTOF MS^1^ of the native ganglioside mixture isolated from secondary brain tumor. Ion source 1: 25.00 kV; ion source 2: 21.30 kV; lens: 10.50 kV; reflector: 26.30 kV; reflector 2: 13.85 kV, pulsed ion extraction: 50 ns. Matrix: 3,5-DHB. Solvent: MeOH.

Purified extracts of secondary brain metastasis tissue were submitted to (−) MALDI QTOF MS analysis in reflector mode using DHB matrix for sample ionization.  Figure 2 illustrates MS^1^ spectrum for the mass range domain of *m/z* 200-2400, which covers the major groups of complex lipid molecular species.

The vast majority of the most abundant mass peaks in Figure 2 corresponds to the sphingolipid class, as follows: *m/z* 521.072, *m/z* 536.072, *m/z* 568.019, *m/z* 618.100, and *m/z* 658.152 correspond to simple ceramides (Cer) with different sphingoid bases (SPB) and fatty acids (FA) in their composition while ions at *m/z* 792.620, *m/z* 806.184, *m/z* 864.709, *m/z* 914.250, and *m/z* 1407.710 are associated with Cer derivatives: Hex-Cer, Lac-Cer, and gangliosides (KDN-GM1). Phosphorylated ceramide structure, CerP 34 : 0; O2 was assigned as [M-H]^−^ for *m/z* 618.100. Cer-1-phosphate has been shown to block apoptosis by inhibiting acid sphingomyelinase and palmitoyl serine transferase in macrophages and also to stimulate cell proliferation by activating phosphatidyl-inositol-3-kinase (PI3K)/ACT [15].

The ion assignment and postulation of structures was carried out by mass calculation and using different lipidomic databases and software (eg. Lipidmaps and Metabolomics Workbench), and is presented in Table 2.

As it can be observed in Table 2, the metastatic brain tissue is rich in simple Cer containing C16–Sphingosine, C18-Sphingosine, C20–Sphingosine, 1-Deoxy–sphinganine, C18-Sphingadiene, C18-Phytosphingosine, C20-Phytosphingosine and a large variety of fatty acids: majority long-chain FA (LCFAs) and very long-chain FA (VLCFAs) with even number of carbon atoms and a few hydroxylated FA: (h14:0), (h16 : 0), (h20 : 0), (h24 : 0), (h26 : 0), and (h32 : 0), some of them with an odd number of carbon atoms, as is the case for (h21 : 0). Most of the FA found in GSLs composition are saturated and a few polyunsaturated VLCFAs.

**Figure 3:**
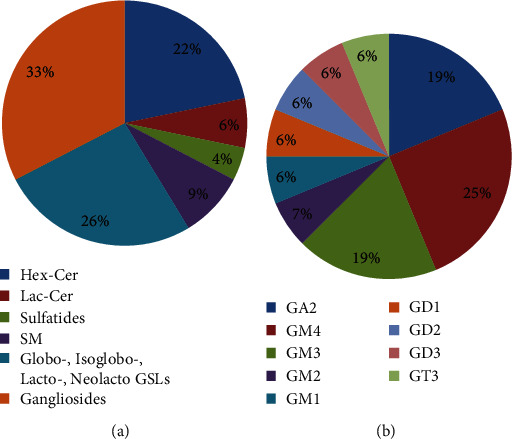
Profile of complex lipids in brain tumor tissue. Relative abundances of (a) GSLs and (b) gangliosides.

However, the lipid profile is dominated by glycolipids, mostly glycosphingolipids (GSL) (Figures 3(a) and 3(b)). Neutral GSL are found as follows: gluco- and galactocerebrosides (Hex-Cer) are associated with ion peaks at *m/z* 714.123, *m/z* 730.094, *m/z* 744.093, *m/z* 766.078, *m/z* 792.620, *m/z* 806.184, *m/z* 864.709, *m/z* 953.470, lactosylceramides (Lac-Cer) at *m/z* 864.709, *m/z* 886.630, *m/z* 914.250, and *m/z* 974.073, and other hexosylceramides at *m/z* 1631.857 which were attributed to type I A antigen (d18:1/20 : 0) having carbohydrate moiety GalNAc *α* 1-3 (Fuc *α* 1-2) Gal *β* 1-3 GlcNAc *β* 1-3 Gal *β* 1-4 Glc *β* or/and type II A antigen with the oligosaccharide chain GalNAc *β* 1-3 (Fuc *α* 1-2) Gal *β* 1-4 GlcNAc *β* 1-3 Gal *β* 1-4 Glc *β* attached to Cer. Asialilated gangliosides of GA2 type are found at *m/z* 1007.031, *m/z* 1077.730, and *m/z* 1108.151 while other neutral GSLs belonging to lacto, neolacto, globo, and isoglobo at *m/z* 1591.939 and *m/z* 1631.857.

**Figure 4:**
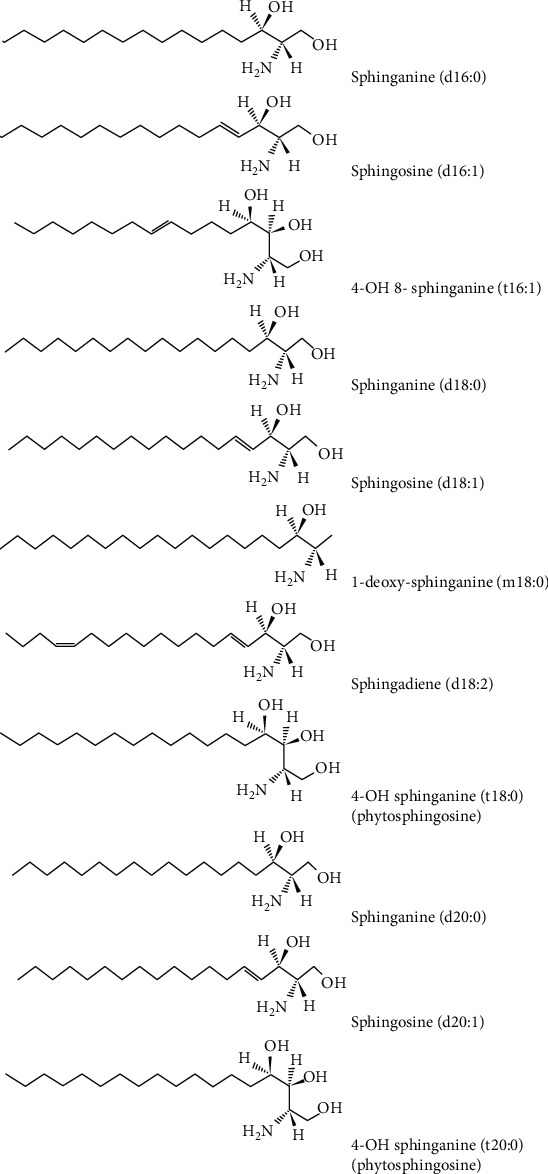
The types of sphingoid bases found in the structure of various GSLs of the brain tumoral mixture.

Acidic GSL are represented by sulfatides found at *m/z* 766.078 and *m/z* 974.073, and GSL species belonging to the ganglioside series. The gangliosides which were identified in the cerebral tumor sample are monosialylated (GM4, GM3, GM2, and GM1), disialylated (GD1, GD2, and GD3) and trisialylated (GT3) structures (Figure 3(b)). The composition of ceramides in the structure of gangliosides is dominated by SPB 18 : 1; O2 and for a few species SPB 18 : 0; O2, SPB 18 : 1; O3, SPB 16 : 1; O2, and SPB 16 : 1; O3 (Figure 4), while the FA vary in length from medium-chain FA (MCFA) (10 : 0, 12 : 0) to VLCFA (32 : 0).

The presence of unusual deaminoneuraminic (KDN) and N-Glycolylneuraminic (Neu5Gc) acids in gangliosides is noticeable in the structures KDN-GM1(t16 : 1/12 : 0) and GalNAc *β* 1-4 (NeuGc *α* 2-3) Gal *β* 1-4 Glc *β*- Cer(d18:1/20 : 0), respectively, which are assigned for [M-H]^−^ ions in MS^1^ found at *m/z* 1407.710 and *m/z* 1426.274. KDN was also found in the following structure KDN *α* 2-3 Gal *β* 1-4 (Fuc *α* 1-3) GlcNAc *β* 1-3 Gal *β* 1-4 Glc *β*-Cer(d18:1/26 : 1) identified at *m/z* 1759.163.

Moreover, the GSL species identified at *m/z* 2286.826 as a minor peak was assigned to carbohydrate ligands of selectins, sLea-x and sLea–Lex with the structure Hex(4)-HexNAc(2)-Fuc(2)-NeuAc-Cer 42 : 1; O2. These antigens are usually not expressed in nonmalignant tissue. In addition to monosialyl Lewis antigens, other GSL which was described to function as a tumor associated antigen [16, 17], disialyl Lea, was identified at *m/z* 2092.106.

Nonglycosilated phosphorylated sphingolipid structures, SM, are assigned only to ion peak at *m/z* 728.120.

### 3.3. MS/MS Analysis of Lipid Species Isolated at *m/z* 1407.710 in Brain Metastatic Tissue

**Figure 5:**
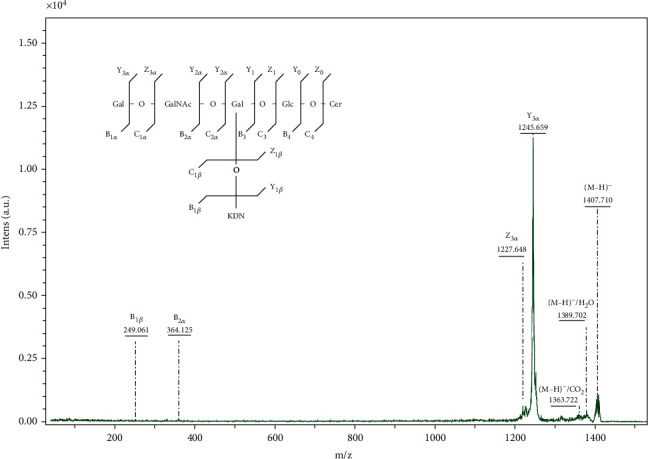
(-) MALDI QTOF MS/MS of the ion [M-H^+^]^−^ at *m/z* 1407.710 isolated from secondary brain tumor. Ion source 1: 25.00 kV; ion source 2: 21.30 kV; lens: 10.50 kV; reflector: 26.30 kV; reflector 2: 13.85 kV, pulsed ion extraction: 50 ns. Matrix: 3,5-DHB. Inset:. Fragmentation scheme of KDN-GM1 (t16 : 1/12 : 0).

To assess the structure of lipid species at *m/z* 1407.710, [M-H^+^]^−^ precursor ion was subjected to “LIFT” mode for tandem MS. The obtained fragmentation ions are presented in Figure 5 and their assignment in Table 3.

The designation of the fragmentation ions resulting from the cleavage of the oligosaccharide chain was made according to the nomenclature of carbohydrate fragmentation introduced by B. Domon and C. Costello [18].

The ion observed at *m/z* 1363.722 results after decarboxylation of deaminoneuraminic acid, attached do the inner Gal (Figure 5, Table 3).

The presence of potassiated KDN residue is supported by the ion at *m/z* 249.061 identified as B_1*β*_, while the ions at *m/z* 1227.648 and *m/z* 1245.659 identified as Z_3*α*_ and Y_3*α*_, respectively, results from KDN-GM1 (t16:1/12 : 0) species after removing of external Gal residue (Figure 5, Table 3).

The attachment of KDN residue at GalNAc instead of inner Gal it is possible in the case of GSLs belonging to the “globo” series, namely of disialyl-Gb5 type ((SA)Gal-(SA)GalNAc-Gal-Gal-GlcCer); however a structure of this type is completely improbable even in the case of a potential in source single cleavage of the terminal sialic acid residue, or accompanied by the cleavage of the outer Gal molecule due to the longer oligosaccharide chain existing in the “globo” series with the typical Di-Gal core attached to Glc-Cer, so that the *m/z* ratio for the fragments that would be obtained would be higher than those in the MS/MS spectrum depicted in Figure 5.

### 3.4. MS/MS Analysis of Lipid Species Isolated at *m/z* 792.620 in Brain Metastatic Tissue

**Figure 6:**
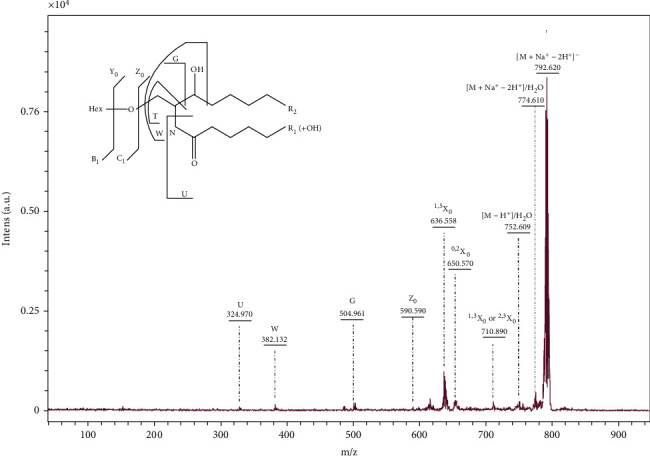
(-) MALDI QTOF MS/MS of the ion [M + Na^+^-2H^+^]^−^ at *m/z* 792.620 isolated from secondary brain tumor. Ion source 1: 25.00 kV; ion source 2: 21.30 kV; lens: 10.50 kV; reflector: 26.30 kV; reflector 2: 13.85 kV, pulsed ion extraction: 50 ns. Matrix: 3,5-DHB. Inset:. Fragmentation scheme of Hex-Cer(d18 : 0/h20:0).

The GSL species detected in MS^1^ at *m/z* 792.620 was subjected to tandem MS analysis by using the “LIFT” mode. The obtained mass spectrum is presented in Figure 6, together with the pathway of fragmentation followed by the precursor ion as an inset.

Table 4 presents the assignment of oligosaccharide sequence ions according to the nomenclature introduced by Domon and Costello [18] and the designation of the fragmentation ions of ceramide by using the nomenclature introduced by Ann and Adams [19].

The presence of Hex residue attached to ceramide is shown by the appearance in the MS/MS spectrum of Z_0_-type ion at *m/z* 590.590 which is formed after elimination of Hex residue together with the X-type ring cleavage ions: ^1,5^X_0_ at *m/z* 636.558, ^0,2^X_0_ at *m/z* 650.570 and ^1,3^X_0_ or ^2,3^X_0_ at *m/z* 710.890.

The monodeprotonated ions at *m/z* 774.610 and *m/z* 752.609 are formed after water elimination from the sodiated and desodiated precursor ion, respectively.

The (d18 : 0/h20:0) ceramide composition is confirmed by the presence of Z_0_–type ion at *m/z* 590.590 and the ions which result from the cleavage of ceramide moiety as follows: G-type ion observed at *m/z* 504.961, which is formed by breaking of C2-C3 bond of the dihydrosphingosine residue, the ion at *m/z* 382.132, which results from further cleavage of the dihydrosphingosine residue at the C3-C4 bond (denoted as W-type), and the U-type fragment ion observed at *m/z* 324.970, which supports the (h20:0) composition of the FA, but without documenting the exact position of the hydroxyl residue.

## 4. Discussion

The lipid composition of cancer cells showed variations in comparison with the normal cell profile, but it also varies between various tumor types [20, 21]. Moreover, there is no specific lipid profile distinctive for tumor cells that would differentiate them from nontumoral ones and this profile may change in time depending on its physiological state. Studies concerning this issue showed that tumor cells are characterized by disorganization of lipids in the membrane and a loss of lipid asymmetry which leads to a decrease in membrane permeability and altered cell signaling [22]. This may be connected to the metabolic reprogramming of tumor cells. Thus, an important alteration related to the length of FA chains and their degree of unsaturation was observed in tumor cells, and this can change the physical properties of the cell membrane, resulting in the increase of membrane fluidity, an effect also observed by reducing the cholesterol content of the membrane in cells preparing for metastasis [22–24]. As an observation consistent with our data, various studies showed that cancer cells keep a lower degree of unsaturation in order to protect plasma membrane lipids from peroxidation [25, 26]. Moreover, an enlarged concentration of saturated FA in the cell membrane lipid bilayer results in improved stability of lipid raft domains and a subsequent better protein binding ability of the cell membrane [25].

Another change in the lipid profile, which can function as a metastatic and antiapoptotic signal, is the elevated cerebroside concentration in the plasma membrane. Additionally, cells with increased cerebroside levels have a tendency to be more chemotherapy resistant than those with normal cerebroside content, probably due to the induction of the mutant p53 expression [27]. GlcCer increased levels and upregulation of GlcCer synthase showed to neutralize downstream apoptosis signals, which are initiated by ceramide and induce a multidrug resistance phenotype in many cancer cells, is one of the most important explanations for the failure of chemotherapy. This relationship supports the association of chemotherapeutic drugs and modulators of Cer metabolism to counteract the depletion of proapoptotic sphingolipids and increase the effectiveness of drugs against cancer [15]. Moreover, the inhibition of GlcCer synthase was shown to delay tumor development of some murine cells [28] and to restore p53-dependent apoptosis in cancer cells carrying p53 deletion mutants [27].

One of the hallmarks of cancer has been acknowledged as being aberrant glycosylation as glycans participate in many cancer-associated events. This alteration often involves sialic acids (SA) which play essential roles in cell-cell interaction, recognition, and immunological response [29]. It has been shown that the metastatic capacity of murine cells in culture was apparently related to their total sialic acid content and to the extent to which sialic acid molecules are exposed on the tumor cell surface, essentially bound to Gal and GalNAc residues present on the cell surface oligosaccharide chains [30, 31].

Thus, distinctive modifications in the sialylation pattern especially for glycolipids have been identified in many transformed tumor cells. An examination of the total serum SA levels in patients with lung cancer, with or without metastases when compared to healthy controls revealed that these were substantially increased [32]. The same observation has been valid also for other tumors associated with metastases [33–36].

SA epitopes are constituents of many cell-surface receptors and have a capacity to mask specific cellular recognition sites involved in the host reaction to external cells, including tumor cells [37]. The masking of immune recognition by NKT cells, through binding of SA on tumor cells to SA-binding immunoglobulin-type lectins (Siglecs), especially to Siglec 7 and Siglec 9, which are found on the surface of immune (NK) cells, may be a significant mechanism of immune escape in malignant tumors as a result of immunosuppressive signals promoted by Siglecs [38–43].

Extensive literature data show that sialylated oligosaccharides of glycolipids have been implicated in tumor progression and metastasis in various types of tumors. Malignant transformation is associated with abnormal glycosylation [44], resulting in synthesis and expression of altered carbohydrate determinants, including terminal fucosylated and sialylated Lewis antigens, sLeA and sLeX. These carbohydrate antigens play a major role in the selectin-mediated adhesion of cancer cells to vascular endothelium, this interaction being the first step of hematogenous metastasis [45–48]. It also has been hypothesized that extensive fucosylation of the serum microenvironment might contribute to the loss of adhesion observed during the process of tumor formation [49].

Expression levels of the structures carrying sLeA and sLeX and of the glycosyltransferases synthesizing sLeA and/or sLeX were also found to correlate with unfavorable outcomes of patients suffering from different tumor types and the occurrence of cancer-associated glycans, sLeX, and sLeA is considered a result of epigenetic silencing of genes involved in GlcNAc 6-sulfation or *α*2-6 sialylation (which results in the expression of normal antigens of the epithelial cells, sialyl 6-sulfo Lewis X, and disialyl Lewis A), event which may affect cancer cell glycans at the relatively early stages of carcinogenesis, but the mechanism is not exactly cancer-specific [50]. Higher expression of sLeA and sLeX in NSCLC tumors has been shown to correlate with an upregulation of fucosyltransferases FUT3 and FUT6. Thus, a promising new therapeutic approach in this direction is represented by hindering selectin–ligand interaction during metastasis by specific drugs [51, 52].

Among the described changes in the lipid composition of malignant cell membranes, particular attention has been given to the notable alterations on the SA containing glycolipids.

Some studies have shown a positive correlation between the degree of sialylation and metastatic capacity of experimental tumors. This was observed for disialylated species, GD3 and GD2 [53–55], and GD3 synthase (GD3S), the regulatory enzyme of GD3 and GD2 synthesis was studied for its potential to serve as a novel drug target in cancer [56]. Moreover, increased expression of disialylated ganglioside species with short-chain, GD3, in brain metastases of pulmonary adenocarcinoma, both in the less aggressive form, as well as in the poorly differentiated, aggressive form, can be associated with the invasive and metastatic character of the primary tumor, as GD3 stimulates adhesion, proliferation, migration, and invasion of tumor cells as well as angiogenesis [56], thus promoting the process of metastasis. However, the monosialylates species GM1, GM2, and GM3 together with GD1a showed to have contrary effects [53–55]. Although several investigations support the role of GM3 in the suppression of cancer development and progression [57, 58], overexpression of NeuGcGM3 has received special attention because it is minimally expressed on most normal human tissues. The absence of Neu5Gc in humans due to deletion in the CMP-N–acetyl-neuraminic acid hydroxylase (CMAH) gene which encodes the hydroxylase converting CMP-Neu5Ac to CMP-Neu5Gc; however Neu5Gc-sialoconjugates have been detected mainly in tumor cells, with their expression attributed to the incorporation of dietary Neu5Gc in the plasma membrane [59]. Exposure to foods containing Neu5Gc (especially red meat) early in life may result in its integration into lipooligosaccharides by commensal bacteria leading to generation of anti-Neu5Gc antibodies (xeno-autoantibodies) against Neu5Gc-containing glycans (xeno-autoantigens); this can lead to xenosialitis, which may have important implications in promoting tumor progression and initiating cancer [59]. While the acetyl substituent of neuraminic acid (Neu5Ac) is predominant under physiological conditions in acidic GSL structures, some tumor cells were shown to produce an excess of gangliosides that contain glycol groups (Neu5Gc) [22, 60]. Thus, many studies revealed that the GM3 (Neu5Gc) ganglioside is expressed only on the tumor cells surface (commonly in lipid rafts) in a large variety of human cancers: in non–small–cell lung cancer (NSCLC), breast cancer melanoma, in malignant epithelial tumors of the digestive tract, in pediatric tumors,, in lymphomas, and in sites of metastases including lymph nodes, in tumors of the kidney, urinary bladder, ovary, uterus, testis, and prostate, in Wilms tumors, other neuroectodermal tumors, sarcomas, and in thyroid carcinomas [61–73].

NeuGc-containing gangliosides, including GM3, were found to be widely expressed in non-small-cell lung cancer (NSCLC), and NeuGc-containing ganglioside expression was associated with patient survival, a lower overall and progression-free survival [61] being associated with a higher expression of Neu5Gc-containing gangliosides, predominantly GM3 (Neu5Gc) as negative prognostic marker. GM3 (Neu5Gc) was rarely identified in the corresponding healthy tissues, confirming the tumor-specific expression of this molecule. The distinction between the effects of GM3 containing NeuGc and GM3 containing NeuAc on the inhibition of EGFR tyrosine kinase might contribute to improvement in the prognosis of these patients [61]. In this view, a monoclonal IgM antibody binding to N-glycolyl-containing gangliosides has been evaluated for a wide range of tumors expressing these particular type gangliosides, including NSCLC showing promising results for the treatment of patients with advanced stage NSCLC [74].

Preferential expression of Neu5Gc by tumor cells is attributed to their higher metabolic rate and induction of the sialin sialic acid transporter by hypoxia. As cancer cells commonly have enhanced cell-surface sialylation, and have increased demand for sialic acid donors for use in the synthesis of sialylated glycans, the uptake and reuse of sialic acids through sialin may save the energy required for their de novo synthesis [75, 76].

Much evidence for the hypothesis that gangliosides play fundamental roles in regulating well-known hallmarks of cancer emerged in the last decades are as follows [77]: (i) involved in sustaining proliferative signaling as the most fundamental trait of cancer cells by modulating growth factor receptor activation or interacting with tumor suppressors; thus, ganglioside GM3 regulate lateral membrane movements via cholesterol and sphingolipid-rich membrane rafts and GM3 (Neu5Gc) interaction with epidermal growth factor receptor (EGFR) in the lipid raft could enhance the signaling through this receptor, promoting tumor progression [77]; (ii) the release of the gangliosides to the tumor microenvironment (ganglioside shedding) has immune suppressive effects; significant amounts of gangliosides with short fatty acid chains in the ceramide portion are spontaneously released in the form of monomers, micelles, and membrane vesicles, allowing insertion into the membranes of host cells, in turn inducing immune suppression [78–81]. GM3(Neu5Gc) interaction with CD4 + T lymphocytes resulted in their insertion in the membrane of T cells, reducing their expression; in addition, GM3(Neu5Gc) similarly down-regulated CD4 expression in CD4 + CD25- T cells and in natural regulatory T cells (CD4 + CD25+), and reduced the proliferative capacity of CD4 + CD25- T cells and the secretion of anti-inflammatory cytokines; moreover, as a consequence of GM3(Neu5Gc) shedding, the differentiation and maturation of murine dendritic cells (DC) was affected [82, 83]. Consistent with this, NSCLC tissues expressing high GM3 (Neu5Gc) have a decrease amount of mature CD83+ DC cells [82, 83]; (iii) anti-Neu5Gc IgG Abs play an important role in tumor immune surveillance, being able to kill tumor cells which express GM3 (Neu5Gc) using complement, and also by a complement-independent oncotic necrosis mechanism. The amount of this Abs in the sera of patients decreases with increasing age, and are often found in very low percentages or absent in the sera of NSCLC patients [78].

In contrast to Neu5Ac and Neu5Gc, KDN (deaminoneuraminic acid) and KDN glycoconjugates are abundant in only lower vertebrates and pathogenic bacteria [84]. However, some data show that the synthesis of free KDN by human cells is enhanced in hypoxia and it occurs in a variety of animal organs and some human lung cancer cells [85, 86].

In mammalian cells and tissues, KDN mostly occurs as the free sugar and little occurred conjugated to glycolipids and/or glycoproteins. It has been postulated that high expression of KDN in mammalian organisms may be thoroughly related to elevated activities of enzymes involved in the formation of sialoglycoconjugates and/or aberrant supply of the precursor sugar, mannose, used in the biosynthesis of KDN [87].

In humans, sialic acid synthase (SAS, NANS) catalyzes the formation of both KDN-9-P and Neu5Ac-9-P using as precursors mannose 6-phosphate and N-acetyl-mannosamine 6-phosphate and hence an increased KDN level could be a reflection of high Neu5Ac synthesis and may result in overexpression of membrane-bound sialoglycoproteins. Although the functions of KDN are still unclear, the conservation of KDN synthase in vertebrates, including humans, suggests physiological relevance yet to be understood [88].

Overexpression of sialic acids, mainly Neu5Ac, on cell surface proteins may possibly change the fate of the cell from normal to malignant. Therefore, the elevated cellular levels of KDN and Neu5Ac might be reflecting the same metabolic consequence [87].

Increased sialylation may also enhance the masking effect of sialic acids on antigenic sites of tumor cells, which turn out to be more like “self” and consequently more invasive. Some studies evidenced that the total concentration of sialic acids in serum of patients suffering of lung cancer was found to be elevated compared to that of healthy controls and, thus, it could be considered as a potential biomarker for lung cancer [89].

Regarding KDN expression, it was observed as being increased in ovarian adenocarcinomas, and positively correlated with the degree of malignancy [86, 90].

Yabu et al. [91] showed that high level of free KDN-containing complex-type N-glycans accumulated in human prostate cancers, while Wang et al. [92] evidenced an elevated level of free KDN comparative to free Neu5Ac and Neu5GC in throat cancers displaying no lymphatic metastasis, and which are poorly to moderately differentiated, and proposed that free KDN may be useful as a biomarker for detecting some early-stage cancers at biopsy, and can be of promising prognostic significance in determining the stage of malignancy. Nevertheless, the mechanism of synthesis of KDN in the tumour cells is still unconvinced to date.

It has been described that under hypoxic conditions free KDN increases in mammalian tumor cells, and it is likely that it supports the hypoxia-resistance property frequent found in tumors [75].

KDN residues have distinct properties comparatively to Neu5Ac and Neu5Gc e.g. are resistant to known bacterial and mammalian sialidases and inhibits the elongation of polysialyl chains by serving as the terminal sugar residue [93, 94].

Oligo/poly(*α* 2,8-KDN) was detectable in the lung of human embryos up to 2 weeks old, and undetectable in the alveolar and bronchial epithelium 3 weeks after birth and in adult human organisms. However, in various histological types of lung carcinomas and cell lines derived therefrom it was reexpressed and represents a valuable oncodevelopmental marker [86]. As polysialylation has an anti-adhesive effect on cell-cell interactions, it is likely to be involved in the detachment and metastasis of cancer cells, although the molecular mechanism is still unclear).

In contrast to Neu5Ac which is abundant in both normal and tumor cells and tissues, the occurrence of KDN is uncommon/unusual and nearly undetectable in normal adult human tissues. Therefore, it is possible that free KDN and glycoconjugates containing KDN may play significant roles in malignant cell growth and could serve as sensitive markers for the occurrence of various human tumors.

In addition, immunotherapeutic approaches targeting other tumor-associated gangliosides (GD3, GM2, and GD2) are being developed; nevertheless, no glycolipid-targeted therapy has yet produced substantial clinical improvements.

Although emerging evidence highlighted that altered lipid composition of the cell membrane is strongly associated with tumorigenesis and tumor progression, the molecular mechanisms involving sialylated glycolipids in primary and secondary brain tumor carcinogenesis have not yet been elucidated. Therefore, further investigations of tumor-associated glycolipid profiles, and particularly of sialylated glycolipids, are to be expected to add new data for the development of diagnostic and therapeutic targets in cancer research.

**Figure 7:**
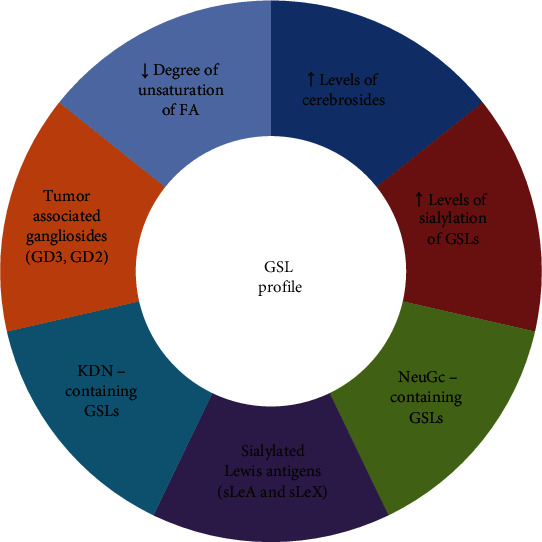
Particularities of GSL profile related to brain tumoral tissue.

In conclusion, glycolipid profile of brain metastasis with NSCLC-type origin showed to be characterized mostly by acidic GSLs, gangliosides, of which GD2 and GD3 were previously conected to the metastatic character of the primary tumor and some species containing unusual deaminoneuraminic and N-Glycolylneuraminic acids, which were showed to be expressed in some human lung cancer cells (Figure 7). The occurence of altered carbohydrate determinants sLeA and sLeX emphasizes the presence abnormal glycosylation as a hallmark of malignant transformation and increased expression of cerebrosides can act in addition as a metastatic and antiapoptotic signature. Glycocalyx composition influences all aspects of tumor cell progression including the metastatic spread, however more data are necessary to quantify the relative contribution of glycolipid components of the glycocalyx in sustaining the procancer phenotype and at the complexity of intrinsic mechanisms of this process in general and at the brain level in particular. This aspect represents a considerable step forward in cancer immunotherapy with antibodies against glycolipids and for other therapies based on tumor-associated carbohydrate antigens.

Being a preliminary study, its main limitation is its small sample size and consequently its statistical power is limited. Nevertheless, the obtained results open up the possibility of more prospective studies on a larger group of patient samples and with other types of primary tumors which evolved with brain metastasis, in order to help elucidate the role of glycosphingolipids in the brain metastasis of different primary tumors.

## Figures and Tables

**Table 1:** Dilutions performed and concentrations obtained for methanolic solutions obtained from the native extract of cerebral metastatic tissue.

No.	Dilution (volumetric ratio)	Concentration after dilution (*μ*g/*μ*L)	Concentration after dilution (pmol/*μ*L)
1	1 : 10	0.354	172.17
2	1 : 20	0.177	86.08
3	1 : 50	0.0708	34.43
4	1 : 100	0.0354	17.21
5	1 : 200	0.0177	8.60
6	1 : 500	0.00708	3.44
7	1 : 700	0.00505	2.45
8	1 : 1000	0.00354	1.72

**Table 2:** Assignment of ions obtained by MALDI TOF MS^1^ screening of brain tumoral lipid mixture.

No.	*m/z*	Ion	Structure
1	390.948	[M-H]^−^	FA 26 : 2 (17,20) or FA 26 : 2 (5,9)
2	521.072	[M-H]^−^	Cer (d18:1/15 : 0)↔Cer 33 : 1; O2
3	536.072	[M-H]^−^	Cer (d18:1/16 : 0) or Cer (d18:0/16 : 1) or Cer (d20 : 1/14 : 0) ↔ Cer 34 : 1; O2
4	554.045	[M-H]^−^	Cer (t18:0/16 : 0)↔Cer 34 : 0; O3
5	568.019	[M-H]^−^	Cer (t18:0/17 : 0)↔Cer 35 : 0; O3
6	590.004	[M-H]^−^	Cer (t18:0/19 : 3)↔Cer 37 : 3; O3
7	606.290	[M-H]^−^	Cer (d18:1/21 : 0) or Cer (d16:1/23 : 0)↔Cer 39 : 1; O2
8	618.100	[M-H]^−^	Cer (d18:2/22 : 0)↔Cer 40 : 2; O2 or Cer (d18:0/16 : 0)–1-P↔CerP 34 : 0; O2
9	640.134	[M-H]^−^	Cer (t18:0/h21 : 0)↔Cer 39 : 0; O4
10	654.127	[M-H]^−^	Cer (t18:0/24 : 6)↔Cer 42 : 6; O3
11	658.152	[M-H]^−^	Cer (t18:0/24 : 4)↔Cer 42 : 4; O3
12	666.217	[M-H]^−^	Cer (t18:0/24 : 0) or Cer (t20:0/22 : 0) or Cer (d20:0/h22 : 0)↔Cer 42 : 0; O3
13	670.121	[M-H]^−^	Cer (t18 : 0/25 : 5)↔Cer 43 : 5; O3
14	672.144	[M-H]^−^	Cer (t18:0/25 : 4)↔Cer 43 : 4; O3
15	692.135	[M-H]^−^	Cer (d18:0/27 : 0)↔Cer 45 : 0; O2 or Cer (d18:1/h26 : 0) ↔ Cer 43 : 1; O3
16	714.123	[M-H]^−^	Hex-Cer (t18:2/h16 : 0)
17	728.120	[M-H]^−^	SM(d18:1/18 : 1) or SM(d18 : 0/18 : 2) or SM(d18:2/18 : 0)
18	730.094	[M-H]^−^	Hex-Cer (t18 : 0/17 : 0)
19	744.093	[M-H]^−^	Hex-Cer (t18:0/18:0) or hex-Cer (d20:0/h16 : 0)
20	762.682^∗^	[M-H]^−^	Hex-Cer(d18:1/21 : 3)
21	766.078	[M-H]^−^	Sulfatides: HSO3-3Gal*β*-Cer (d18 : 1/h14:0) or Hex-Cer (d18:2/21:0)
22	776.036	[M-H]^−^	Cer (d18:1/h32 : 0)↔Cer 50 : 1; O3
23	792.620	[M + Na^+^ − 2H^+^]^−^	Hex-Cer (d18:1/h20:0)
24	798.594	[M-H]^−^	Hex-Cer (d18:1/24 : 6)
25	806.184	[M-H]^−^	Hex-Cer (t18:0/23 : 4)
26	864.709	[M + K^+^ − 2H^+^]^−^ [M-H]^−^	Hex-Cer (t18:0/24 : 1) O-ac-lac-Cer (d18:1/12 : 0)
27	886.630^∗^	[M-H]^−^	Lac-Cer (d18:1/18 : 1)
28	914.250	[M-H]^−^	Lac-Cer (d18:1/20 : 1)
29	921.595^∗^	[M-H]^−^	GM4 (d16 : 0/13 : 0)
30	953.470	[M + 2Na − 3H]^−^	GM4 (d18:0/10 : 0)
31	974.073	[M-H]^−^	Sulfatides: HSO3-3Gal*β*-Cer (d18:1/30 : 0) Or lac-Cer (d18:0/24 : 0)
32	1007.031	[M-H]^−^	GA2 (d18:1/12 : 0)
33	1077.730	[M-H]^−^	GA2 (t18:1/16:1)
34	1108.151	[M-H]^−^	GA2 (d18:1/h18:0)
35	1129.836	[M-H]^−^	GM4(d18:1/26 : 0)
36	1181.756	[M-H]^−^	GM3 (d18:0/18 : 0)
37	1215.049	[M-H]^−^	GM4(d18:0/32:0)
38	1407.710	[M-H]^−^	KDN-GM1(t16:1/12 : 0)
39	1426.274	[M-H]^−^	GalNAc*β*1-4(NeuGc*α*2-3)Gal*β*1-4Glc*β*-Cer(d18:1/20 : 0)
40	1466.801	[M + Na − 2H]^−^	GD3 (d18:0/16 : 0)
41	1467.243	[M-H]^−^	GM2 (d18:1/24 : 0)
42	1516.941	[M-H]^−^	GM1 (d18:1/16 : 0)
43	1544.947	[M-H]^−^	GM1 (d18:1/18:0)
44.	1591.939	[M-H]^−^	Ganglio:Gal*β*1-4GalNAc*β*1-3Gal*β*1-3GalNAc*β*1-4Gal*β*1-4Glc*β*-Cer(d18:1/16:0) (Ganglio series) Lacto:Gal*β*1-3GlcNAc*β*1-3Gal*β*1-3GlcNAc*β*1-3Gal*β*1-4Glc*β*-Cer (d18:1/16 : 0) (lacto series) Neolacto: Gal*β*1-4GlcNAc*β*1-3Gal*β*1-4GlcNAc*β*1-3Gal*β*1-4Glc*β*-Cer (d18:1/16 : 0)(Neolacto series) Globo: GalNAc*α*1-3Gal*β*1-3GalNAc*β*1-3Gal*α*1-4Gal*β*1-4Glc*β*-Cer (d18:1/16 : 0)(Globo series) Isoglobo: Gal*α*1-3(GalNAc*β*1-4Gal*β*1-4GlcNAc*β*1-6) Gal*β*1-4Glc*β*-Cer (d18:1/16 : 0)(Isoglobo series)
45.	1631.857	[M-H]^−^	Type I A antigen: GalNAc*α*1-3(Fuc*α*1-2)Gal*β*1-3GlcNAc*β*1-3Gal*β*1-4Glc*β*-Cer(d18:1/20 : 0)(lacto series) Type II A antigen: GalNAc*β*1-3(Fuc*α*1-2)Gal*β*1-4GlcNAc*β*1-3Gal*β*1-4Glc*β*-Cer(d18:1/20 : 0) Or GalNAc*α*1-3(Fuc*α*1-2)Gal*β*1-4GlcNAc*β*1-3Gal*β*1-4Glc*β*-Cer(d18:1/20 : 0)(Neolacto series) Globo:Fuc*α*2-3GlcNAc*β*1-6GalNAc*β*1-3Gal*α*1-4Gal*β*1-4Glc*β*-Cer(d18:1/20 : 0) (Globo series) Isoglobo: Fuc*α*1-3GlcNAc*β*1-3Gal*α*1-3(GalNAc*β*1-4) Gal*β*1-4Glc*β*-Cer(d18:1/20 : 0)(Isoglobo series)
46.	1758.206	[M-H]^−^	GD2(d18:1/24 : 0)
47.	1759.163	[M-H]^−^	KDN*α*2-3Gal*β*1-4(Fuc*α*1-3)GlcNAc*β*1-3Gal*β*1-4Glc*β*-Cer(d18:1/26 : 1)
48.	1873.273	[M-H]^−^	GT3(d18:1/26 : 0)
49.	2092.106	[M-H]^−^	Fuc-GD1b(d18:1/26 : 1) or Disialyl lea (d18:1/26 : 1): NeuAc*α*2-3Gal*β*1-3 (NeuAc*α*2-6) (Fuc*α*1-4) GlcNAc*β*1-3Gal*β*1-4Glc*β*-Cer(d18:1/26 : 1)
50.	2194.225	[M-H]^−^	GalNAc*β*1-4(NeuAc*α*2-3)Gal*β*1-3GlcNAc*β*1-3(Gal*β*1-3GalNAc*β*1-4)Gal*β*1-4Glc*β*-Cer(d18:1/24 : 1)
51.	2286.826	[M-H]^−^	sLea -Lex: NeuAc*α*2-3Gal*β*1-3(Fuc*α*1-4)GlcNAc*β*1-3(Gal*β*1-4(Fuc*α*1-3)GlcNAc*β*1-6)Gal*β*1-4Glc*β*-Cer(d18:1/24 : 0) or sLea-x: NeuAc*α*2-3Gal*β*1-3(Fuc*α*1-4)GlcNAc*β*1-3Gal*β*1-4(Fuc*α*1-3)GlcNAc*β*1-3Gal*β*1-4Glc*β*-Cer(d18:1/24 : 0)

FA: fatty acid; Cer: ceramide; Gal:Galactose; Glc:Glucose, Lac: Lactose; Ac: acetyl; SM: sphingomyelins; NeuAc: N-acetylneuraminic acid; KDN: 2-keto-3-deoxy-D-glycero-D-galacto-nononic acid; NeuGc; N-Glycolylneuraminic acid.

**Table 3:** Assignment of fragmentation ions obtained in MS^2^ from the precursor ion [M-H^+^]^−^ at *m/z* 1407.710.

Fragment no.	*m/z*	Assignment of the fragmentation ions
1	1363.722	[M-H^+^]^−^/CO_2_
2	1389.702	[M-H^+^]^−^/H_2_O
3	1227.648	Z_3*α*_
4	1245.659	Y_3*α*_
5	364.125	B_2*α*_
6	249.061	B_1*β*_

**Table 4:** Assignment of fragmentation ions obtained in MS^2^ from the precursor ion at *m/z* 792.620.

Fragment no.	*m/z*	Assignment of the fragmentation ions
1	324.970	U
2.	382.132	W
3	504.961	G
4	590.590	Z_0_
5	636.558	^1,5^X_0_
6	650.570	^0,2^X_0_
7	710.890	^1,3^X_0_ or ^2,3^X_0_
8	752. 609	[M-H^+^]/H_2_O
9	774.610	[M + Na^+^ − 2H^+^]/H_2_O

## Data Availability

All data used to support the findings of this study is included within the article.
